# Identification and Biochemical Characterization of Protein Phosphatase 5 from the Cantharidin-Producing Blister Beetle, *Epicauta chinensis*

**DOI:** 10.3390/ijms141224501

**Published:** 2013-12-16

**Authors:** Xi’en Chen, Shumin Lü, Yalin Zhang

**Affiliations:** Key Laboratory of Plant Protection Resources and Pest Management of Ministry of Education, Northwest A&F University, Yangling 712100, China; E-Mails: chenpp2006@nwsuaf.edu.cn (X.C.); shuminlv@nwsuaf.edu.cn (S.L.)

**Keywords:** *Epicauta chinensis*, protein phosphatase 5, *E. coli* expression, *in vitro* inhibition

## Abstract

Protein phosphatase 5 (PP5) is a unique member of serine/threonine phosphatases which has been recognized in regulation of diverse cellular processes. A cDNA fragment encoding PP5 (*EcPP5*) was cloned and characterized from the cantharidin-producing blister beetle, *E. chinensis. EcPP5* contains an open reading frame of 1500 bp that encodes a protein of 56.89 kDa. The deduced amino acid sequence shares 88% and 68% identities to the PP5 of *Tribolium castaneum* and humans, respectively. Analysis of the primary sequence shows that *EcPP5* has three TPR (tetratricopeptide repeat) motifs at its *N*-terminal region and contains a highly conserved *C*-terminal catalytic domain. RT-PCR reveals that *EcPP5* is expressed in all developmental stages and in different tissues. The recombinant EcPP5 (rEcPP5) was produced in *Escherichia coli* and purified to homogeneity. The purified protein exhibited phosphatase activity towards pNPP (p-nitrophenyl phosphate) and phosphopeptides, and its activity can be enhanced by arachidonic acid. *In vitro* inhibition study revealed that protein phosphatase inhibitors, okadaic acid, cantharidin, norcantharidin and endothall, inhibited its activity. Further, protein phosphatase activity of total soluble protein extract from *E. chinensis* adults could be impeded by these inhibitors suggesting there might be some mechanism to protect this beetle from being damaged by its self-produced cantharidin.

## 1. Introduction

Reversible phosphorylation of structural and functional proteins catalyzed by kinases and phosphatases is associated with the control of a wide variety of intracellular processes [1]. The numbers of catalytic subunits of protein phosphatases is nearly an order of magnitude lower than protein kinases [2]. Protein phosphatases are structurally and functionally diverse enzymes that comprise three major families: phosphoprotein phosphatases (PPPs), metal-dependent protein phosphatases (PPMs), and the aspartate-based phosphatases represented by FCP/SCP. The PPP family includes protein phosphatase 1 (PP1), PP2A, PP2B (or calcineurin), PP4, PP5, PP6 and PP7. These PPP family members are among the most highly conserved proteins known [3,4].

In contrast to other PPP family members comprising isoforms encoded by different genes, PP5 is encoded by a single gene. Another unique structural characteristic of PP5 is that it possesses a highly conserved catalytic Ser/Thr phosphatase domain at the *C*-terminal half of protein as well as three to four tetratricopeptide repeat (TPR) domains at its *N*-terminal extension. These domains are believed to be involved in protein-protein interactions and activation of PP5 via specific binding to unsaturated fatty acids [5,6]. Unlike most of the PP1 and PP2A, purified PP5 exerts a low basal activity [7]. In mammals, PP5 regulates intracellular biological activities as diverse as cellular proliferation, differentiation, migration, survival and death, and DNA damage repair [8]. A recent study revealed that PP5 has an important role in the signaling mechanism associated with the diclazuril-induced merozoite apoptosis in *Eimeria tenella* [9]. To date, our knowledge of insect PP5 is still limited.

The blister beetles (Coleoptera: Meloidae) and some of the smaller oedemerid beetles (Coleoptera: Oedemeridae) are perhaps the most notorious of all poisonous insects due to their defensive toxin, cantharidin, which has been recognized as a potent PPPs inhibitor [10,11]. Mammal PP5 has been identified to be sensitive to several natural toxins, such as okadaic acid, microcystins, nodularin, calyculin A, tautomycin and cantharidin [12]. So far, there is no corresponding knowledge about whether insect PP5 is sensitive to these toxins. In addition, it is useful to investigate whether PP5 or other PPPs in cantharidin-producing beetles can be inhibited by cantharidin.

Herein, we undertook a study towards the identification and characterization of PP5 from a cantharidin-producing beetle, *Epicauta chinensis*. We analyzed the expression of this gene and the properties of its protein product along with its sensitivity to some PPPs inhibitors *in vitro*.

## 2. Results and Discussion

### 2.1. Cloning and Sequence Analysis

So far, PP5 genes from several insect species have been identified and published in the NCBI Database (National Center for Biotechnology Information, U.S. National Library of Medicine, Bethesda, MD, USA). However, no further study has analyzed their sequences or determined their function in biological processes. From our previously established transcriptome database of *E. chinensis* adults, we obtained the fragment containing the entire *EcPP5* ORF of 1500 bp, which encodes a protein of 499 amino acids with the predicted molecular mass of 56897.92 Da and theoretical isoelectric point of 5.91. The nucleotide sequence of *Ec*PP5 cDNA was deposited in the GenBank (NCBI) database with the accession number KF765498.

**Figure 1.**
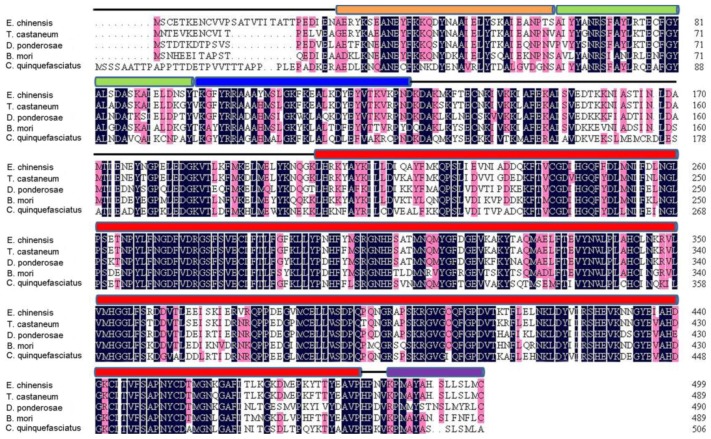
Amino acid sequence comparison of insect Protein phosphatase 5 (PP5s). Alignment of the deduced amino acid sequence of *Ec*PP5 was made with other insect PP5s from *Tribolium castaneum* (accession number XP 971407), *Dendroctonus ponderosae* (accession number AEE62915), *Bombyx mori* (accession number XP 004923376), and *Culex quinquefasciatus* (accession number XP 001850926). The positions of the three TPR (tetratricopeptide repeat) domains are indicated with lines of orange, green, and blue above the sequence. The catalytic domain and helix αJ motif are indicated with a *red line* and *purple line* above those sequences, respectively.

**Figure 2.**
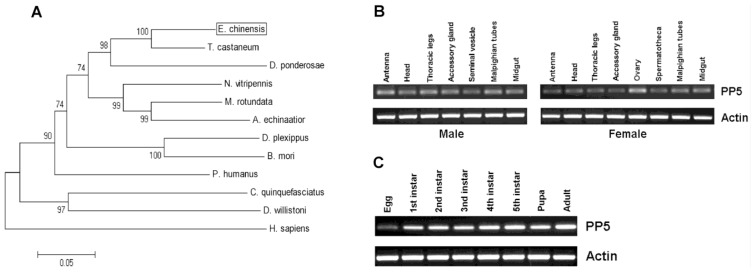
Phylogenetic tree construction and expression analysis of *EcPP5*. (**A**) Phylogenetic analysis of *EcPP5* with other insect PP5s; (**B**) RT-PCR analysis of *EcPP5* expression in different tissues of male and female adults; and (**C**) RT-PCR analysis of *EcPP5* expression in various developmental stages. The actin gene (*EcActin*) was used as the reference gene.

Prosite analysis indicated the presence of three TPR (tetratricopeptide repeat) domains at its *N*-terminal portion which also exists in other known PP5s (Figure 1). Structural analysis of human PP5 revealed that access to the active site of the phosphatase domain is blocked by its TPR domains which maintains the low basal activity of PP5 [13]. The *C*-terminus consists of the highly conserved phosphatase domain containing three characteristic sequence motifs within the PPP family, GDxHG, GDxVDRG, and GNHE which play important roles in metal coordination, substrate binding and catalysis [4,14,15]. It also carries a nuclear targeting domain which is responsible for its transport into the nucleus. Additionally, a helix αJ motif was found at its *C*-terminus which could strengthen the association between the TPR domains and the phosphatase domain of PP5 [13] (Figure 1). BLAST analysis of *EcPP5* deduced an amino acid sequence on NCBI that revealed a strong similarity with PP5s from other insect species with the highest identity being 88% to the PP5 of *T. castaneum*. It also shares a 68% identity to human PP5. The constructed phylogenetic tree shows the PP5 of *E. chinensis*, *T. castaneum*, and *D. ponderosae* from Coleoptera form a small cluster (Figure 2A).

### 2.2. Tissue- and Stage-Specific Expression Patterns of *EcPP5*

Tissue-specific expression patterns of *EcPP5* gene were analyzed in seven different tissues from male adults and eight different tissues from female adults, respectively, by using RT-PCR. Our results indicated that the *EcPP5* gene was expressed in all tissues examined. In males, the expression levels among different tissues were nearly the same. While in the female, the *EcPP5* transcript is more abundant in the ovary than in other tissues (Figure 2B). This may be the first report of tissue-specific expression of insect PP5.

Stage-specific expression patterns of the *EcPP5* gene were determined for eggs, five different larval instars (1st, 2nd, 3rd, 4th and 5th), pupae and adults by using RT-PCR. We found that PP5 was expressed in all stages, with the lowest expression in eggs and the highest expression in adults (Figure 2C). In *D. melanogaster*, PP5 was expressed across the life cycle, but more highly expressed in the embryonic than at later developmental stages [16].

The transcript of *EcPP5* was detectable in different tissues and developmental stages, suggesting that PP5 might participate in many biological processes in *E. chinensis*. However, more research will be needed to understand its role in biological activities in this beetle or other insects.

### 2.3. Expression and Purification of Recombinant *EcPP5*

Usually, expression of active PPP-type phosphatases in *E. coli* is difficult. *E. coli*-expressed PPP-type phosphatases are generally incorrectly folded and inactive. To facilitate their correct folding, chaperons, such as GroEL/GroES, have been used [17]. Since metal ions are indispensable for their phosphatase activity, a low concentration of metal ions (usually 2 mM MnCl_2_) needs to be externally added to the culture media [5]. PP5 genes from mammal, *Plasmodium falciparum*, and *Trypanosoma brucei* have been successfully expressed in *E. coli* [7,18,19]. However, there is no report for the heterologous expression of insect PP5.

**Figure 3.**
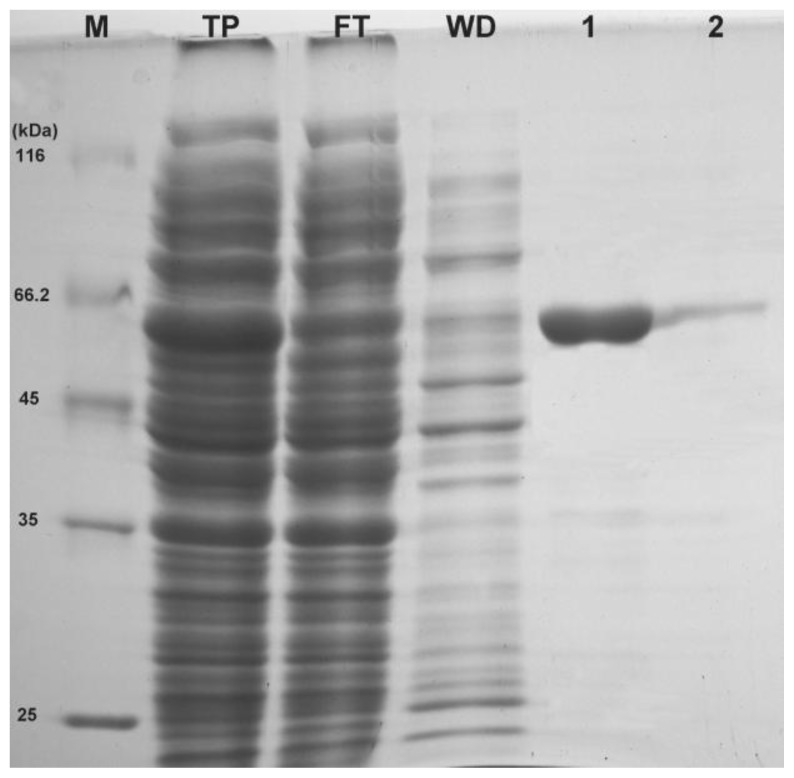
Purification of rEcPP5. **M**: protein marker; **TP**: total soluble cell lysate; **FT**: flow-through elution; **WD**: wash-down elution; **1**: 50 mM imidazole elution; **2**: 150 mM imidazole elution.

Herein, the *EcPP5* ORF was subcloned into a modified expression vector pET43.1b and transformed into *E. coli* BL21(DE3)plysS cells for production of recombinant EcPP5 (rEcPP5). The *EcPP5* gene was expressed as inclusion bodies in *E. coli* under 1.0 mM IPTG (isopropyl β-D-thiogalactoside), at 37 °C (data not shown), whereas, highly soluble rEcPP5 was produced under 0.1 mM IPTG, at 18 °C, with 2 mM Mn^2+^ incorporated in the LB media. As expected, a band with an apparent molecular weight of ~60 kDa was detected on 12% SDS-PAGE (Figure 3) which is close to the predicted protein size from the deduced amino acid sequences. The soluble *N*-terminal His_6_ tagged rEcPP5 was collected by a single step of affinity chromatography using Ni-NTA resin (Transgen, Beijing, China). High purity rEcPP5 protein (>90%) was eluted by 50 mM imidazole (Figure 3). After being dialyzed overnight at 4 °C, the protein concentration was determined. Finally, ~1.8 mg of highly purified rEcPP5 was obtained from 500 mL cell culture.

### 2.4. Biochemical Characterization of the rEcPP5 Protein

**Figure 4.**
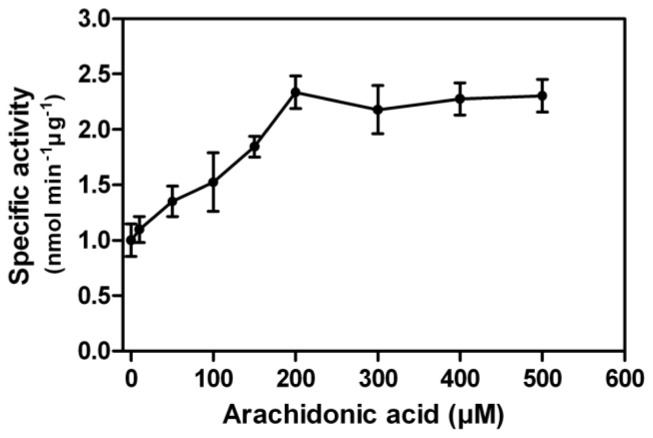
Activation of rEcPP5 by arachidonic acid. Each point is the mean ± SD of three independent assays.

In contrast to other PPP family members, a unique property of PP5 is the stimulation of its phosphatase activity by unsaturated fatty acids. Using pNPP as the substrate, we found arachidonic acid stimulated the rEcPP5 activity up to 2.3-fold in a dose dependent manner with the maximum activation occurring at 200 μM (Figure 4). This stimulation result was similar with those found on *E. coli* expressed *Trypanosoma brucei* PP5, 2.6-fold [18], and *Plasmodium falciparum* PP5, 2.0-fold [19]. It has been reported that the activation of mammalian PP5 activity by arachidonic acid varied from 4 to 25-fold [7,20]. We considered that the conformation of rEcPP5 expressed in *E. coli* might differ somewhat from the native one resulting in an increased intrinsic activity. We also carried out the arachidonic acid activation assay against total crude protein phosphatase extracted from *E. chinensis* adults. Total extracts showed phosphatase activity towards phosphopeptides, yet could not be stimulated by arachidonic acid (data not shown), which is probably due to the very low abundance of PP5 in the cells, or the activity is inhibited by intrinsic cantharidin. It is still notoriously difficult to purify the native PP5 to investigate whether arachidonic acid could stimulate its activity to higher folds.

**Figure 5.**
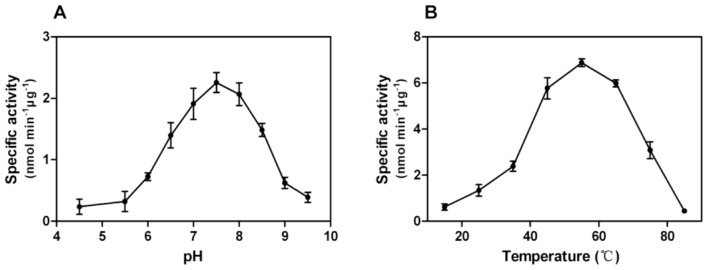
(**A**) Optimum reaction pH; and (**B**) optimum reaction temperature determination of rEcPP5. Each point is the mean ± SD of three independent assays.

The optimal reaction pH or temperature was determined from a series of reactions in different pH assaying buffer or in pH 7.4 assaying buffer at varying temperatures. The phosphatase activity of rEcPP5 first increased and then declined with ascending pH or temperature. The optimum reaction pH is about 7.4 (Figure 5A) and the optimum reaction temperature is around 55 °C (Figure 5B). The underlying mechanism for rEcPP5 possessing high phosphatase activity at relatively high temperatures is unknown. We presumed that rEcPP5 might undergo conformational changes with the increasing temperature which makes the substrate more easily accessible to its active site, while at a higher temperature its conformation begins to be destroyed leading to the loss of activity. So far, knowledge about the activity of PP5 *in vivo* at high temperatures is limited.

### 2.5. Detection of rEcPP5 Phosphatase Activity *in Vitro*

**Figure 6.**
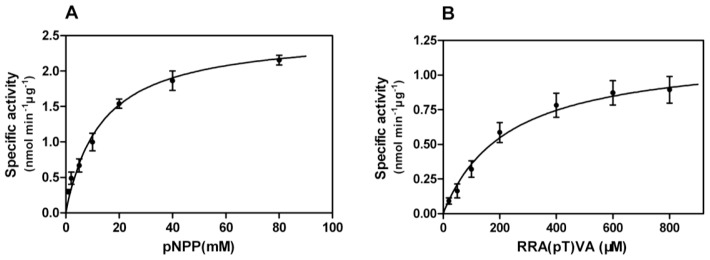
Michaelis-Menten plots of rEcPP5 toward pNPP (**A**) and phosphopeptides (**B**). Each point is the mean ± SD of three independent assays.

Enzyme-substrate reactions were carried out and kinetic properties were determined from Michaelis-Menten plots (Figure 6A,B), yielding *Km* of 13.32 mM, *Vmax* of 2.54 nmol min^−1^·μg^−1^ towards pNPP, and *Km* of 227.1 μM and *Vmax* of 1.17 nmol min^−1^·μg^−1^ against phosphopeptides. *kcat* and *kcat*/*Km* values were calculated as 2.54 s^−1^ and 0.19 mM^−1^·s^−1^ for pNPP and 1.17 s^−1^ and 5.15 mM^−1^·s^−1^ for phosphopeptides, respectively. Phosphopeptides have proved to be more specific substrates than pNPP for PPP-type phosphatases [21], and our results of lower *Km* and higher *kcat*/*Km* values also indicate that phosphopeptides are more specific and exert a stronger affinity to rEcPP5; however, we consider pNPP more qualified as a substrate in our assays. The reasons are as follows: (1) the method for using pNPP in the assays is simple to set up and easy to carry out; (2) the maximal catalytic activity of pNPP is usually much higher than that of phosphopeptides and can often approach that of a natural substrate [21]; and (3) the purified protein was taken as the enzyme source in our assays which circumvented the interaction between pNPP and other unexpected phosphatases.

### 2.6. Inhibition of Compounds on r*EcPP5* and Crude PPP Extract

**Figure 7.**
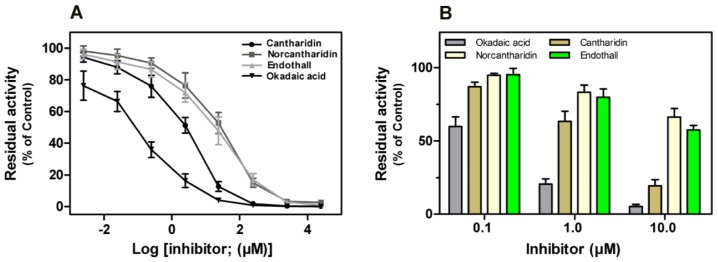
Inhibition assay of compounds on rEcPP5 (**A**) and on PPP extract (**B**). Each point is the mean ± SD of three independent assays.

Previous studies revealed that some naturally occurring toxins, such as okadaic acid, cantharidin, *etc.*, could potently impair the phosphatase activity of both native and recombinant mammalian PP1, PP2A, PP4 and PP5 *in vitro* [12]. Here we found all four compounds exerted potent inhibitory effect on rEcPP5 in a dose-response response (Figure 7A). Okadaic acid inhibited rEcPP5 activity with an *IC*_50_ value of 0.19 μM that is significantly higher than the value reported for native bovine PP5 (*IC*_50_ = 3.5 nM). However, the sensitivity of rEcPP5 to cantharidin, *IC*_50_ = 3.19 μM, was similar to recombinant mammal PP5 sensitivity to cantharidin (*IC*_50_ = 3.5 μM) [12]. The *IC*_50_ values of norcantharidin and endothall were 25.69 and 28.41 μM, respectively. There has been no report concerning the inhibitory effect of norcantharidin and endothall on PP5 before. Further study is needed to determine the sensitivity of native EcPP5 to these inhibitors. It will be of interest to compare the differences in the inhibitor-sensitivity between insect PP5 and mammal PP5.

It has been reported that cantharidin and endothall inhibited PPPs activity in the total soluble protein extract from *Arabidopsis thaliana* and *Lemna paucicostata* [22]. These four compounds showed an inhibitory effect on PPP activity in the extract of *E. chinensis* (Figure 7B). Okadaic acid was found to be the most potent inhibitor, with the residual phosphatase activity of only 8.79% at 10 μM. Cantharidin potently inhibited phosphatase activity in the extract, with the residual phosphatase activity of 19.36% at 10 μM. The inhibition of PPP activity by norcantharidin seemed as effective as inhibition by endothall, with no significant differences in the residual phosphatase activity at each concentration. This result is in agreement with the above results on the inhibitory effect of these compounds on rEcPP5.

We found cantharidin, together with the other three known PPP inhibitors, could block the activity of *E. coli-*expressed recombinant PP5 and PPP in the total protein extract from a cantharidin-producing beetle, *E. chinensis.* Given the highly conserved catalytic domain of the PPP family across the Eukaryota, it seems like that other members of PPP in *E. chinensis* are sensitive to cantharidin and other PPP inhibitors. Consequently, a question arises: how does this beetle prevent its own cantharidin accessing and affecting on its endogenous PPP? Work is currently in progress to answer this question.

## 3. Experimental Section

### 3.1. Insects and Chemicals

Adults of the blister beetle *E. chinensis* were collected at their eclosion peak from soybean fields in Suide County, Shaanxi Province, China, on July 2011. All adults were kept in a chamber (50 cm × 50 cm × 50 cm) supplied with fresh lucerne (*Medicago sativa*) or soybean (*Glycine max*) leaves at 28.0 ± 2.0 °C, with a 14L:10D photoperiod. The larvae were reared individually in 150 mL plastic cups and fed with locust (*Locusta migratoria*) eggs under conditions of 30.0 ± 1.0 °C, 10.0 ± 1.0% soil humidity, and a photoperiod of 16L:8D.

Okadaic acid, cantharidin, arachidonic acid, and pNPP were purchased from Sigma Chemical Corporation (St. Louis, MO, USA). Norcantharidin and endothall was purchased from Alfa Aesar Chemical Co. Ltd. (Haverhill, MA, USA). All other chemicals were of research grade or better and were obtained from commercial sources.

### 3.2. Identification, Cloning, and Sequence Analysis of *PP5* from E. chinensis

The *EcPP5* gene fragment was identified by searching the sequences in our previously established transcriptome database for *E. chinensis* adults via using the *PP5* gene sequence of *Tribolium castaneum* (accession: XM_966314) as a probe. The putative *EcPP5* fragment was further searched using BLASTX (NCBI) against the non-redundant database at NCBI to confirm its identity as a *PP5* gene.

The open reading frame (ORF) of *EcPP5* was predicted via ORF Finder (NCBI). Primers for amplification of the ORF of *EcPP5* were a sense primer 5′-CATATGCACCACCACCACCACCACAGTTGTGAAACGAAAGAG-3′ with *Nde* I restriction site (underlined) and 6 histidine codons (italic), and an antisense primer 5′-CTCGAGCTAGCACATTAGACTTAGTAAAG-3′, containing the *Xho* I restriction site (underlined). Total RNA was extracted from adults using Trizol Plus (TaKaRa, Dalian, China) according to manual instructions. The quality and concentrations of RNA samples were examined by agarose gel electrophoresis and spectrophotometer analysis. RNA was digested with DNase I (TaKaRa, Dalian, China) and cDNA was synthesized using M-MLV RNaseH^−^ reverse transcriptase (Fermentas, Ontario, Canada) and stored at −20 °C until use. PCR reaction was carried out on the S1000™ Thermal Cycler (BioRad, Philadelphia, PA, USA) using ExTaq (TaKaRa, Dalian, China) under the following conditions: 95 °C for 3 min, 30 cycles of 95 °C for 30 s, 60 °C for 30 s, 72 °C for 1.5 min, followed by a final extension at 72 °C for 7 min. The purified PCR products were inserted into pMD 19-T vector (TaKaRa, Dalian, China) and transformed into *E. coli* DH5α (TaKaRa, Dalian, China), then sequenced (AuGCT, Inc., Beijing, China).

The amino acid sequence of EcPP5 was deduced from the obtained ORF cDNA sequence and the similarity analysis on the deduced amino acid sequence was performed using BLAST programs. The phylogenetic tree was constructed by MEGA 5.0 [23] by the maximum parsimony method using amino acid sequences of other insect PP5s, *Tribolium castaneum* (XP 971407), *Dendroctonus ponderosae* (AEE62915), *Megachile rotundata* (XP 003699533), *Nasonia vitripennis* (XP 001603324), *Acromyrmex echinatior* (EGI70292), *Danaus plexippus* (EHJ67807), *Pediculus humanus* (XP 002425763), *Bombyx mori* (XP 004923376), *Culex quinquefasciatus* (XP 001850926), and *Drosophila willistoni* (XP 002070260). The human PP5 (NP 006238) was employed as an outgroup. The ExPASy Compute pI/*M*w tool [24] was used to predict the molecular weight and isoelectric points of EcPP5. For the analysis of protein domains, the InterProScan 4 from EMBL-EBI [25] was used to search the InterPro collection of protein signature databases.

### 3.3. Stage- and Tissue-Dependent Expression Analysis

For stage-specific expression study, eggs, 1st–5th instar larvae, pupae and adults were collected and stored at −80 °C until use. For tissue-specific expression, antenna, head, thoracic legs, accessory glands, seminal vesicles, Malpighian tubules, and midgut were dissected from male adults on ice and stored at −80 °C until use. Antenna, head, thoracic legs, accessory glands, ovary, spermatheca, Malpighian tubules, and midgut were dissected from females on ice and stored at −80 °C until use. Total RNA was extracted as mentioned above. cDNA was synthesised from 1.0 μg total RNA using a PrimeScript RT reagent kit with gDNA eraser (TaKaRa, Dalian, China). The forward primer, 5′-AAACGTGGTGTTGGTTGTCA-3′, and the reverse primer, 5′-TTCCATCGTGAGCAATTTCA-3′, were designed using Primer 3 [26]. The *E. chinensis β-actin* gene (accession: JQ764814) was used as a reference for RT-PCR analysis, with the forward primer, 5′-GCACCTGAAGAACATCCA-3′, and the reverse primer, 5′-ACCAGAAGCATACAACGA-3′. Using 100-fold diluted cDNAs as templates, RT-PCR reactions were performed by a thermal cycle programme consisting under the following conditions: 95 °C for 3 min, 28 cycles of 95 °C for 30 s, 60 °C for 1 min, and a final extension at 72 °C for 7 min. The intensities of PCR products on 1% agarose gel were detected using a BioRad Gel Doc 2000 system (BioRad, Hercules, CA, USA). RT-PCR reaction was repeated at least 3 times, each with a new preparation of total RNA.

### 3.4. Expression and Purification of Recombinant EcPP5

The open reading frames (ORF) of EcPP5 with 6 histidine codons and *Nde I* site at *N*-terminal, and *Xho I* site at *C*-terminal, were cloned into *Nde I* and *Xho I* (TaKaRa, Dalian, China) treated *pET43.1b* expression vector (Novagen, Madison, WI, USA), and transformed into *E. coli* BL21(DE3)plysS cells (Novagen, Madison, WI, USA). The positive transformed cells were amplified in LB medium containing 4 mM Mn^2+^, 100 μg/mL ampicillin and 34 μg/mL chloramphenicol. Recombinant EcPP5 (rEcPP5) expression was induced with 0.1 mM isopropyl β-D-thiogalactoside (IPTG) when *A*_600_ reached 0.4–0.6 and this was continued for 24 h at 18 °C.

Cells were harvested by centrifugation at 7000× *g* for 10 min 4 °C and sonication for 5 min on ice in Buffer A (20 mM Tris-HCl, pH = 8.0, 20 mM imidazole, 300 mM NaCl, 4 mM MnCl_2_) containing 0.1% β-mercaptoethanol, 1.0 mg/mL isozyme and 1 mM PMSF (phenylmethanesulfonyl fluoride). Debris was pelleted by centrifugation (20,000× *g* for 20 min at 4 °C). The soluble fraction was loaded onto a Buffer A pre-equilibrated Ni-NTA affinity column (Transgen, Beijing, China) to bind the target protein; the non-target proteins were washed down with Buffer B. The target protein was eluted with 5 column volumes of Buffer A containing series concentrations of imidazole. Eluted solutions were subjected to 12% SDS-PAGE and stained with Coomassie Blue (Sigma-Aldrich, St. Louis, MO, USA). The rEcPP5 containing fractions were pooled and dialyzed against Buffer B (Buffer A + 50% glycerol) overnight and then stored at −20 °C. Protein concentration was assessed using the method of Bradford [27].

### 3.5. Crude PPP Extraction

The frozen adults were ground into a homogeneous state in ice-cold 20 mM Tris (pH = 7.4), 0.1% β-mercaptoethanol, 1 mM EDTA, 1 mM benzamidine and 1 mM (PMSF). This was then centrifuged at 12,000× *g* for 30 min at 4 °C. The supernatant extract was desalted using Sephadex G-25 spin columns (GE Healthcare, Boston, MA, USA) to remove the contaminating free phosphate as described in the instruction manual. Protein concentration was estimated using the Bradford method [27].

### 3.6. Determination of Phosphatase Activity

Activity of rEcPP5 against the inorganic phosphatase substrate pNPP was determined as described with slight modifications [28]. Assays were performed at 30 °C in a 100 μL reaction containing 500 ng purified rEcPP5, 200 μM arachidonic acid and 20 mM pNPP in 20 mM Tris (pH = 7.4), 1 mM EDTA, 1 mM EGTA, 0.1% β-mercaptoethanol and 0.1% ethanol. After being pre-warmed to 30 °C, the reaction was initiated by adding the pNPP. Assays were terminated after 15 min with 100 μL of 5 N NaOH. Non-enzyme samples were taken as controls. Sample absorbance was measured at *A*_410_ on Infinite^®^ 200 PRO multimode micro-plate reader (Tecan, Austria). Kinetic parameters of rEcPP5 were estimated by using a Michaelis-Menten plot analysis of data obtained under the above assay conditions with pNPP concentrations of 1, 2, 5, 10, 20, 40 and 80 mM.

Phosphatase activity of rEcPP5 was also determined towards phosphopeptides using PPP assay kit (Promega, Madison, WI, USA) in half-area 96-well plates (Corning Inc., New York, NY, USA) following the manual’s instructions. In brief, 500 ng protein was mixed in an assay buffer of 50 mM imidazole (pH = 7.2), 200 μM arachidonic acid, 0.2 mM EGTA, 0.02% β-mercaptoethanol, 0.1 mg/mL BSA, and pre-warmed to 30 °C. Reaction was initiated after adding 100 μM phosphopeptides, RRA(pT)VA to obtain a final volume of 50 μL and incubated at 30 °C for 15 min. The reaction was stopped by adding 50 μL of molybdate dye/additive mixture and incubated for an additional 15 min at room temperature for color development. Control samples were determined without the enzyme. Sample absorbance was measured at A_600_ on an Infinite^®^ 200 PRO multimode micro-plate reader (Tecan, Austria). Kinetic parameters of enzymes were estimated by using a Michaelis-Menten plot analysis of data obtained under the above assay conditions with phosphopeptide concentrations of 20, 50, 100, 200, 400, 600 and 800 μM. All assays were performed for three replications.

PPP activity in the extracts was measured using a PPP assay kit (Promega, Madison, WI, USA) as described above.

Using pNPP as substrate, the optimum reaction temperature of rEcPP5 protein was assayed for different temperatures and optimum reaction pH was determined in various pH substation solutions.

### 3.7. Inhibition Assay

Four candidate inhibitors were dissolved in dimethyl sulphoxide (DMSO) to provide stock solutions of 10 mM for okadaic acid, 100 mM for cantharidin, 200 mM for norcantharidin and endothall, respectively. All solutions were diluted to the desired concentrations with assay buffer before use. Assays were carried out using pNPP as substrate. Before starting the reaction, the enzyme was incubated with the chemicals for 5 min at room temperature. A dose-response assay was used to determine the *IC*_50_. The non-enzyme reaction was taken as the background control, while the non-inhibitor reaction was used as the full-activity control. An inhibition ratio was calculated as the percentage of *A*_410_ values of the inhibition assay reaction divided by the full-activity control, having subtracted the background control *A*_410_ value for both.

## 4. Conclusions

We isolated the *PP5* gene from *E. chinensis* which shows a high sequence similarity with *PP5* from other insects. The transcript of *EcPP5* was detectable in different tissues of adults, as well as in all developmental stages, with the lowest level in eggs and highest level in adults. Soluble recombinant EcPP5 was obtained using an *E. coli* expression system with the addition of Mn^2+^ in the media. The purified rEcPP5 displayed phosphatase activity towards two substrates, pNPP and phosphopeptides. Moreover, similar to PP5 from other organisms, its activity could also be stimulated by arachidonic acid. Four known protein phosphatase inhibitors: okadaic acid, cantharidin, norcantharidin and endothall strongly inhibited rEcPP5 activity. We also found PPP activity in the total soluble protein of extract from this cantharidin-producing beetle could also be blocked by these four PPP inhibitors indicating there might be some protection mechanism to prevent this beetle from being damaged from its self-produced cantharidin.
